# Non-small cell lung cancer: quantitative phenotypic analysis of CT images as a potential marker of prognosis

**DOI:** 10.1038/srep38282

**Published:** 2016-12-06

**Authors:** Jiangdian Song, Zaiyi Liu, Wenzhao Zhong, Yanqi Huang, Zelan Ma, Di Dong, Changhong Liang, Jie Tian

**Affiliations:** 1 Sino-Dutch Biomedical and Information Engineering School, Northeastern University, Liaoning, Shenyang, 110819, China; 2 The Key Laboratory of Molecular Imaging, Institute of Automation, Chinese Academy of Sciences, Beijing 100190, China; 3 Department of Radiology, Guangdong General Hospital, Guangdong Academy of Medical Sciences, 106 Zhongshan Er Road, Guangzhou, 510080, China; 4 Guangdong Lung Cancer Institute, Guangdong General Hospital, Guangdong Academy of Medical Sciences, 106 Zhongshan Er Lu, Guangzhou, 510080, China; 5 Beijing Key Laboratory of Molecular Imaging, Beijing, 100190, China

## Abstract

This was a retrospective study to investigate the predictive and prognostic ability of quantitative computed tomography phenotypic features in patients with non-small cell lung cancer (NSCLC). 661 patients with pathological confirmed as NSCLC were enrolled between 2007 and 2014. 592 phenotypic descriptors was automatically extracted on the pre-therapy CT images. Firstly, support vector machine (SVM) was used to evaluate the predictive value of each feature for pathology and TNM clinical stage. Secondly, Cox proportional hazards model was used to evaluate the prognostic value of these imaging signatures selected by SVM which subjected to a primary cohort of 138 patients, and an external independent validation of 61 patients. The results indicated that predictive accuracy for histopathology, N staging, and overall clinical stage was 75.16%, 79.40% and 80.33%, respectively. Besides, Cox models indicated the signatures selected by SVM: “correlation of co-occurrence after wavelet transform” was significantly associated with overall survival in the two datasets (hazard ratio [HR]: 1.65, 95% confidence interval [CI]: 1.41–2.75, p = 0.010; and HR: 2.74, 95%CI: 1.10–6.85, p = 0.027, respectively). Our study indicates that the phenotypic features might provide some insight in metastatic potential or aggressiveness for NSCLC, which potentially offer clinical value in directing personalized therapeutic regimen selection for NSCLC.

Non-small cell lung cancer (NSCLC) remains the number one cause of cancer-related mortality in the US, and its prevalence continues to increase worldwide^1^. Despite potentially curative resection in early-stage NSCLC, survival remains sub-optimal and recurrence rates are high^2^,^3^. Extracting more prognostic information from the pre-therapy radiological images as the new non-invasive prognostic biomarker for NSCLC is extremely valuable for clinicians.

Personalized medicine is a goal in modern cancer therapy that aims at treating each patient based on the specific tumor characteristics of his/her disease. Evidence has been accumulating suggesting that quantitative image descriptors may yield additional predictive and prognostic information, which could be potentially served as non-invasive prognostic biomarkers for individual disease prognosis^4^,^5^. Comprehensive phenotypic characteristics with valuable clinical meaning can be extracted from radiological images by post-processing techniques. The field of “radiomics” is a further step towards personalized medicine, focusing on the relationship between quantitative biological features and cancer prognosis by non-invasive method, therefore aiding clinicians in selecting the appropriate treatments. It indicates that easily obtainable non-invasive pre-therapy imaging prognostic biomarkers that allow assessment of NSCLC are worth to study^6^,^7^.

As a non-invasive imaging method, computed tomography (CT) has been widely available, and easily used for tumor prognostic evaluation^8^,^9^. Tumor heterogeneity, which described by quantitative intratumoral features, can be assessed in a user-defined region of interest (ROI) on CT images. It includes texture to quantify the spatial pattern or arrangement of pixel intensities, spatial descriptors to measure the sphericity or asymmetry, and voxel-based methods to characterize the uniformity of pixel distribution.

Quantitative methods to measure tumor heterogeneity have been shown to play a role in the assessment of cancer response to therapy^10^,^11^,^12^. Intratumor heterogeneity measured by texture parameters on non-enhanced and/or contrast material-enhanced CT images between baseline and initial post-therapy have been associated with overall survival (OS) in patients with colorectal cancer^13^, metastatic renal cell cancer^14^, esophageal cancer^15^, and NSCLC^16^,^17^,^18^,^19^. More recently, another related research has shown that, as a prognostic radiomics signature, well defined and reproducible texture features were able to separate patients into better survival groups with statistical significance^20^.

The identification of imaging phenotypic signatures with prognostic ability has been increasingly realized^13^,^14^,^16^,^21^, however, to date studies investigating the potential relationships of quantitative phenotypic features with histopathology and clinical TNM staging are still insufficient. The aim of our study was to elucidate the association between quantitative phenotypic features (processed on the pre-therapy CT images) and histopathology (squamous cell carcinoma (SqCC) or adenocarcinoma (ADE)), clinical TNM staging (N0/N1 or N2/N3, T1/T2 or T3/T4, I/II or III/IV), and further evaluated the relationship with OS in patients with NSCLC.

## Results

### Patients

The demographic and tumor characteristics of patients were summarized in Table 1. Of the 661 patients, 545 had ADE (mean age: 60.2 years, SD: 11.3), 116 SqCC (mean age: 61.6 years, SD: 9.1). As for the aggregate TNM groups, 539 patients were included in the T1/T2 group (mean age: 60.6 years, SD: 11.0), 122 in the T3/T4 group (mean age: 59.5 years, SD: 10.5), 507 in the N0/N1 group (mean age: 61.1 years, SD: 10.9), and 154 in the N2/N3 group (mean age: 58.6 years, SD: 11.1). Four hundred and thirty-nine patients had stage I/II (mean age: 61.3 years, SD: 10.6), whereas 222 had stage III/IV disease (mean age: 58.4 years, SD: 11.3). 138 patients were included in the OS analysis (mean age: 60.4 years, SD: 11.2 median survival: 20.5 months). Of the 61 patients on the validation dataset, forty one patients had stage I/II (median survival: 32.7 months), whereas seventeen had stage III/IV disease (median survival: 15.9 months). Demographics and clinicopathologic characteristics of the patients for OS analysis in the primary and TCIA datasets were listed in Table 2.

### Tumor Segmentation

An ad-hoc analysis was performed to evaluate the segmentation accuracy of the automatic lesion segmentation approach we used in this study. Among the 661 patients in primary dataset and the 61 patients in validation dataset, 50 NSCLC patients were randomly selected for the analysis. The average dice coefficient (DC) of the segmentation by radiologists and the results by automatic approach is 81.06%. All the tumors were segmented by the automatic approach and then reviewed by the radiologists, and there are 39 patients were re-segmented by the radiologists.

### Phenotypic signatures for prediction

**Figure 1:**
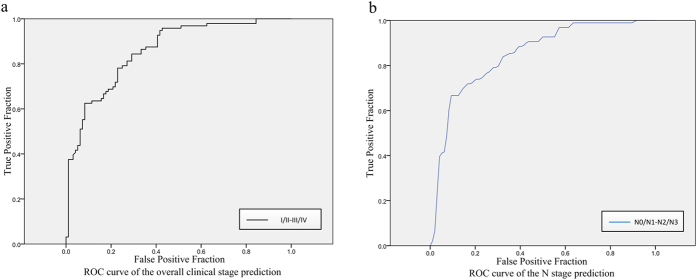
The receiver operating characteristic curves of (**a**) overall clinical stage (stage I/II vs. stage III/IV) and (**b**) N stage (N0/N1 vs. N2/N3) prediction when using the 25 features which are at top of the score list by support vector machine. The area under curves are 0.84 and 0.79, respectively.

Results from the SVM analysis showed that the prediction precision tend to be stable with certain features, as shown by the ROC curves in Fig. 1. The phenotypic features with predictive (“T” represented significantly correlated) and prognostic (HRs and P value by univariate Cox analysis) value were presented in Table 3, respectively. The features: run-length and skewness (skewness in the HL image), which been chosen to predict histopathology (SqCC vs. ADE) achieved 75.16% prediction accuracy. The performance of representative descriptors on N staging (variance of Gabor, run-length and CO-correlation) was 79.40%. And the phenotypic characteristics selected to estimate overall clinical stage (compactness, energy of RL, and sphericity) showed 81.33% prediction precision. Please refer to Table 3 for more detailed statistic information of these classified features. Predictive performances and the ROC curves were presented in Supplemental Material, Appendix A4.

### Phenotypic signatures for prognosis

The prognostic ability of the highest scored features selected by SVM was evaluated by univariate Cox regression model on the primary cohort, which has been shown in the last column of Table 3. Among all these highest scored features selected by the SVM, the feature: LH-CO-correlation (HR: 1.60; 95% CI: (1.13, 2.28); P = 0.004), and HL-CO-correlation (HR: 1.54; 95% CI: (1.08, 2.19); P = 0.016) were significantly associated with OS. The average of Inter-class correlation coefficients (ICCs = 0.833) indicated that there is no significant difference between the signatures from the two datasets.

**Figure 2:**
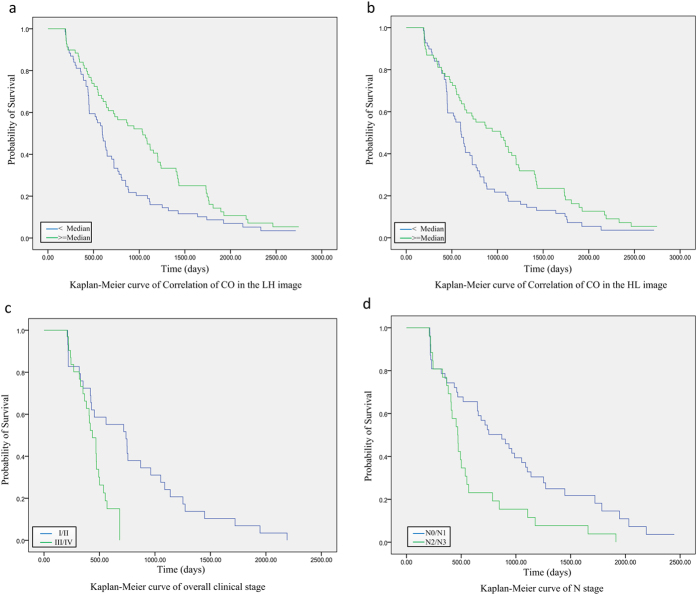
Prognosis performance of the prognostic imaging features. Graph shows results of Kaplan-Meier analysis of survival time for the specified value of (**a**) Correlation of co-occurrence in LH image (P = 0.010) and (**b**) HL image (P = 0.007) on the primary cohort. And graph shows results of Kaplan-Meier analysis of survival time for the specified value of (**c**) overall clinical stage (P = 0.043) and (**d**) N stage (P = 0.010) on the Primary cohort.

After adjustment for age, sex, smoke, clinical TNM staging, pathology, the results from multivariate Cox model indicated that overall clinical stage (I/II vs. III/IV; HR: 0.50; 95% CI: (0.39, 0.85) P = 0.043), N staging (N0/N1 vs. N2/N3; HR: 0.52; 95% CI: (0.31, 0.87) P = 0.010), LH-CO-correlation (HR: 1.65 for low vs. high, 95% CI: (1.41, 2.75); P = 0.010) and HL-CO-correlation (HR: 1.75 for low vs. high; 95% CI: (1.15, 2.58); P = 0.007) were still significantly and independently associated with OS, as shown in Table 4. Figure 2 illustrated the Kaplan-Meier curves of the representative signatures and clinical staging on the primary cohort.

**Figure 3:**
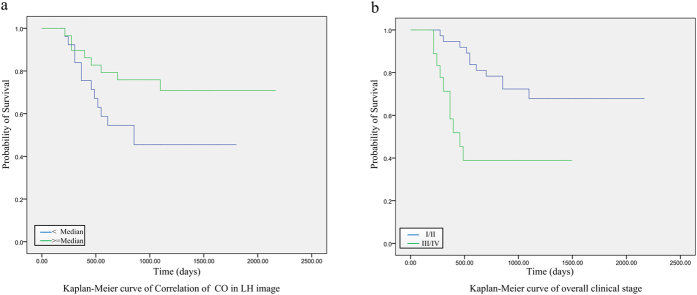
Prognosis performance of the prognostic imaging features. Graph shows results of Kaplan-Meier analysis of survival time for the specified value of (**a**) Correlation of co-occurrence in LH image (P = 0.027) and (**b**) overall clinical stage (P = 0.006) on the validation cohort.

The prognostic performance of the correlation of co-occurrence was further verified on the external validation dataset. Results by multivariate Cox model on the TCIA dataset showed that the patients with lower expression of LH-CO-correlation, the hazard increased by 2.74 times (P = 0.027), which strengthened the conclusion from the primary dataset that patients with high values of LH-CO-correlation indicating better survival probability (HR: 2.74 for low vs. high; 95% CI: (1.10, 6.85); P = 0.027). In addition, according to the TCIA dataset the risk of patients with overall clinical stage III or IV increased more than 3 times compared with the patients of I or II stage (N0/N1 vs. N2/N3; HR: 0.30, 95% CI: (0.12, 0.69), P = 0.006), as shown by the Kaplan-Meier curves in Fig. 3.

## Discussion

This study demonstrated that quantitative phenotypic features from the pre-therapy CT images in patients with NSCLC, not only possessing predictive ability to histology subtype (between SqCC and ADE) and clinical TNM staging (T1/2 vs. T3/4, N0/1 vs. N2/3; aggregated stage I/II vs. stage III/IV), but also can be served as prognostic biomarker to survival. According to the predictive performances and ROC curves, the prediction results for NSCLC tended to be stable when using a certain amount of representative signatures. Results from univariate and multivariate Cox models on the pre-therapy CT images denoted that the feature of CO-correlation is significantly associated with OS in the primary cohort from Asia. This finding has been further verified on the TCIA public dataset from the United States, which strengthened the population-specific strength of the phenotypic signatures proposed in this study for clinical. The patients with higher value of CO-correlation indicating better OS (P = 0.010 in the primary cohort and P = 0.027 in the validation cohort), independent of the effects of other factors. This finding potentially offers clinical value in directing personalized therapeutic regimen selection for NSCLC patients.

From the results of the prediction and prognosis trials, this study potentially provided a way for disease estimation by the proposed radiomics approach. This view was further strengthened by Coroller’s study^22^ on the distant metastasis prediction of lung adenocarcinoma and Grove’s study^23^ on the lung adenocarcinoma prognosis. Compared with those studies, our findings extended the universal of prognostic imaging biomarker on different datasets. Besides, our study indicated that these different clinical stages would be represented differently on CT images. Prediction of clinical stage by intratumor phenotype is a new perspective, which potentially a harbinger that the phenotypic image signatures may provide some insight in metastatic potential or aggressiveness. In addition, this study also confirmed that N stage (N0/N1 vs. N2/N3) and overall clinical stage (I/II vs. III/IV) were significantly related with OS (P < 0.05). However, the difference of T stage (T1/T2 to T3/T4) was not significant (P > 0.05). This might result from the Cox model may not predict the survival well using the T stage alone, but with the interaction of T, N and M stages, the overall clinical stage could describe the prognosis of OS with better precision (P < 0.05)^24^, as previously reported.

Phenotypic descriptors from CT images have been demonstrated that measurements of tumor heterogeneity were potentially associated with glucose metabolism^15^, angiogenesis^17^, and tumor hypoxia^18^. Our study further supported the conception that quantitative measurement of tumor heterogeneity based on the pre-therapy CT images can be associated with the prognosis in NSCLC patients. The texture “correlation of co-occurrence” is a metric of the relationship between marginal probabilities and standard deviation of the co-occurrence matrix, which is a marker indicating the significantly disparity among intratumor voxels, has been verified significantly associated with OS not only on the primary clinical dataset but also be verified on the public dataset from the United States. Although the tumor size, skewness and other statistical characteristics of tumor have been confirmed as prognostic factors of NSCLC^6^, the clinical application of tumor heterogeneity, measured by texture from contrast-enhanced CT images, might provide more information serving as reliable pre-therapy noninvasive imaging biomarker for clinical aided diagnosis.

Several limitations of this study should be addressed in the future. First, because of the complexity of following therapeutic regimens, the treatment after resection was not discussed in this study. A more stratified study should be done to verify the prognostic ability of the proposed phenotypic signatures in different treatments, which may assist for future studies on the prognosis of NSCLC^25^. Next, SVM was the only method used to perform the signature selection; there may be other correlates for phenotypic features that have not been included in our study. Finally, as the contrast-enhanced CT image was the only imaging modality we used, a comparative study of phenotypic features on the different imaging modalities should be developed in the future.

In conclusion, tumor heterogeneity quantified by CT phenotypic signatures may indirectly reflect tumor prognosis. The prognostic imaging biomarkers could be served as harbinger of histology subtype and clinical TNM staging, and overall survival. Results in our study suggest that further research on quantitative image phenotypic features is warranted, with more advanced applications of CT images used for treatment monitoring, outcome prediction, or imaging biomarkers. Identification of poor prognosis by non-invasive methods may help avoid unnecessary drug toxicity and cost, allowing more accurate choice of an alternative treatment regimen that might improve clinical outcome^26^. Since effective and credible clinical aided diagnosis is important to plan subsequent definitive treatment, quantitative radiomics-related studies could provide better prognostic regimen for patients with NSCLC.

## Methods

### Patient population

This retrospective study was approved by our Institutional Review Board (approval #: 2015192H) and waived the requirement for informed consent. The patients who were diagnosed as NSCLC with available surgical tumor samples (aspiration biopsy for patients with advanced stage and lobectomies, segmentectomy or mediastinal nodal biopsies for the early phase) between May 2007 and July 2014 were retrospectively enrolled in our protocol. All CT scans were obtained prior to resection, and the interval between CT scans and resection was one month. A detailed flowchart of this study was presented in Supplemental Fig. S1.

#### Phenotypic signatures for clinical prediction

The inclusion criteria were as follows: (a) age of 18 years or older; (b) pathological diagnosis of NSCLC and contrast-enhanced CT imaging of the chest (c) available date of CT scanning and of the last follow-up time (death or censored). The exclusion criteria were as follows: (a) diagnosis of other diseases over the period of the study; (b) incomplete baseline information, and (c) failure to retrieve clinical diagnosis and/or post-treatment CT studies for central review. The study cohort consisted of a total of 661 patients. Demographic and tumor characteristics of all the patients are summarized in Table 1.

#### Phenotypic signatures for overall survival

Secondly, in order to determine the prognostic ability of CT phenotypic signatures on NSCLC patients, we further validated the association between the key signatures selected by SVM and overall survival, based on the enrollment in the pervious section, the patients who only met the following criteria were selected for the OS analysis: (a) at least six months follow-up and the first follow-up was before January 2013; (b) sustained follow-up visits after surgery; (c) patients who occurred the endpoint event (dead from NSCLC) and (d) continued review after surgery at our institution. One hundred and thirty-eight patients were eligible for the OS analysis (censored cases were withdrawn from this study to ensure accuracy). OS time was defined as the time from the start of resection until the date of death. In addition, other useful baseline clinical variables (tobacco use, recurrence and tumor location) were also gathered from the electronic medical records database.

To evaluate the population-specific strength of the signatures proposed in the overall survival analysis, an independent external validation cohort^23^ from the United States was used in this study. A complete NSCLC dataset which consisted of 61 patients with diagnostic contrast-enhanced CT images were accessed from the cancer imaging archive (TCIA). The CT images of the independent validation cohort are contrast-enhanced CT and the protocol was approved by the Institutional Review Board (IRB). The signatures for survival analysis were then normalized in the two datasets respectively given the different CT protocols^27^. The signatures for survival analysis were normalized into [0, 1] according to the values in each cohort, and the median of signature was used to distinguish the low-level and high-level. The low-level status equals 0 and high-level status equals 1 for Cox proportional hazards regression analysis. A weight log-rank test (the G-rho rank test, rho = 1) was applied to evaluate the survival curves of the high-risk and low-risk groups according to the prognostic signatures^28^. The Kruskal-Wallis H test was applied to these signatures from the two datasets. The ICC was used to access the agreement of the two datasets. The pre-surgery diagnostic CTs obtained within two month, which is a little longer than the primary cohort (one month). Demographic and clinicopathologic characteristics information of the validation dataset from the TCIA database is shown in Table 2.

### CT imaging

All images of the in-house dataset were acquired in the Department of Radiology at our hospital. Contrast-enhanced CT were performed on every patient using one of the two multi-detector row CT (MDCT) systems (GE Lightspeed Ultra 8, GE Healthcare, Hino, Japan or 64-slice LightSpeed VCT, GE Medical systems, Milwaukee, Wis), with the following acquisition parameters: 120 kV; 160 mAs; 0.5- or 0.4-second rotation time; detector collimation: 8 × 2.5 mm or 64 × 0.625 mm; field of view, 350 × 350 mm; matrix, 512 × 512. After routine non-enhanced CT, contrast-enhanced CT was performed after 25 s delay following intravenous administration of 85 ml of iodinated contrast material (Ultravist 370, Bayer Schering Pharma, Berlin, Germany) at a rate of 2.5–3.0 ml/s with a pump injector (Ulrich CT Plus 150, Ulrich Medical, Ulm, Germany). CT image was reconstructed with standard kernel, with interval: 1 mm–2.5 mm. Retrieval of CT images: All of the CT images were retrieved from the picture archiving and communication system (PACS) (Carestream, Canada). The final clinical stage of disease was determined with histological staging and/or surgical evidence of advanced or metastatic disease. T-stage, N-stage, M-stage and final clinical staging were performed as per the AJCC guideline, 7^th^ edition^29^.

### Tumor extraction and analysis

#### Tumor delineation

A complete region of interest (ROI) of lung tumor should be delineated for quantitative phenotypic analysis. We used an automatic segmentation method^30^ in this study, based on the region growing and multi-scale constraints, the method has been evaluated on the Lung Image Database Consortium-Image Database Resource Initiative (LIDC-IDRI) dataset^31^, Manual segmentation would be performed by radiologists once the evaluation of automatic segmentation results was poor by the radiologists in the review stage. Some segmentation results were presented in Supplemental Fig. S2.

#### Feature analysis

A complete NSCLC phenotypic feature set which covered volume, texture, Gabor and wavelet features was extracted on the segmented pre-therapy contrast-enhanced CT images. We then selected the phenotypic signatures with prognostic ability selection method^32^ from the feature set. The meaning of abbreviations, computational formulas and stability analysis of each feature were shown in Supplemental Material, Appendix A1. An independent reproducibility evaluation of the feature extraction was performed by two radiologists. The radiologists were mainly responsible for manual segmentation. All the segmentation of the two observers was preformed in double-blind. The Kruskal-Wallis H test was performed in the reproducibility assessment experiment to assess the differences between the phenotypic features generated per-reviewers with R software version 3.2.3. The consistency of the features from different readers was presented in Supplemental Material, Appendix A2. The support vector machine (SVM) model to find representative features we used in this paper has been widely applied for classification in different fields^33^,^34^,^35^. Each feature was ranked according to its predictive ability on the training dataset by a 5-fold cross-validation process (Detail information of the 5-fold cross-validation was presented in Supplemental Material, Appendix A3). Higher score indicated better predictive performance. The informative, reproducible, and independent phenotypic signatures were selected as the representative signatures for potential prognostic image biomarkers analysis.

### Statistical analysis

Descriptive statistics were expressed as numbers and percentages for categorical variables and “mean ± standard deviation” or medians for continuous variables. In the prediction section of histopathology and clinical TNM staging, the features selected by SVM to distinguish different lung cancer pathologies between SqCC and ADE, early or advanced cancer stage (aggregated overall clinical stage: I/II vs. III/IV; aggregated TNM staging: T1/T2 vs. T3/T4; N0/N1 vs. N2/N3) were assessed by multiple response receiver operating characteristic curves (ROC). Since the M1 staging was considered to be advanced phase (IV staging), this study did not discuss the M staging in particular. ROC curve was defined to measure the fraction of true positive predictions as a function of the fraction of true negative predictions. The curves of prediction of histopathology, TNM and overall clinical staging were exclusively generated by a 5-fold cross-validation process.

Sensibility of the change of those signatures which selected in the previous section on patient’s prognosis was evaluated independently. Cox proportional hazards models were created on the primary dataset (138 patients) and the validation dataset (61 patients) to assess the independent effects of the signatures on OS. We used the median of each feature as the threshold level to dichotomize patients for Cox analysis^5^. Multivariate Cox model was adjusted for age, sex, smoke, histopathology, tumor location and clinical stage. Log-rank tests were performed for the comparisons of the Kaplan-Meier survival curves between groups. All the statistical analyses were performed by the PASW Statistics 18.0.0 (SPSS Company) and R software version 3.2.3, and the results from Cox analysis were reported as hazard ratios (HRs), with 95% confidence intervals (CIs) and P values. Two-sided P values less than 0.05 were considered as a significant difference.

## Additional Information

**How to cite this article**: Song, J. *et al*. Non-small cell lung cancer: quantitative phenotypic analysis of CT images as a potential marker of prognosis. *Sci. Rep.* **6**, 38282; doi: 10.1038/srep38282 (2016).

**Publisher's note:** Springer Nature remains neutral with regard to jurisdictional claims in published maps and institutional affiliations.

## Supplementary Material

Supplementary Materials

## Figures and Tables

**Table 1:** Patient demographics and clinical characteristics for the classification of histopathology and clinical TNM staging.

Demographic or Clinicopathologic Characteristic	Values					
	No. of patients	Age (Y)*	Gender(M)^†^	Tobacco use^†^	Relapse^†^	Side (Left)^†^
**Pathology**						
ADE	545	60.2 ± 11.3	313 (57)	268 (49)	155 (28)	215 (39)
SqCC	116	61.6 ± 9.1	111 (95)	73 (63)	29 (25)	61 (53)
**TNM Staging**						
T1/T2	539	60.6 ± 11.0	364 (68)	192 (36)	144 (27)	245 (45)
T3/T4	122	59.5 ± 10.5	90 (74)	85 (70)	52 (43)	56 (46)
N0/N1	507	61.1 ± 10.9	372 (73)	191 (37)	107 (21)	239 (47)
N2/N3	154	58.6 ± 11.1	97 (63)	91 (60)	81 (53)	68 (44)
M0	586	60.7 ± 10.7	422 (72)	235 (40)	155 (26)	277 (47)
M1	75	58.4 ± 12.8	45 (60)	25 (33)	44 (59)	28 (37)
**Overall clinical Stage**						
I/II	439	61.3 ± 10.6	317 (72)	162 (37)	84 (19)	211 (48)
III/IV	222	58.4 ± 11.3	143 (64)	159 (71)	117 (53)	91 (41)
**Overall Survival**	138	60.4 ± 11.2	80 (58)	49 (36)	62 (45)	45 (33)

Note.— ^†^Data in parentheses are percentages.

*Data are expressed as mean ± standard deviation.

Abbreviations: Ade, adenocarcinoma; SqCC, squamous cell carcinoma.

**Table 2:** Patient demographics and clinicopathologic characteristics of the primary cohort and the validation cohort for overall survival analysis.

Demographic or Clinicopathologic Characteristic	Primary Cohort			Validation Cohort*		
	No. of patients	OS (months)		No. of patients	OS (months)	
		Median	95% CI		Median	95% CI
**Sex**						
Male	83	21.5	(19.5, 30.2)	—	—	—
Female	55	19.6	(17.5, 32.4)	—	—	—
**Age**, **years**						
<65	80	29.9	(18.6, 34.1)	—	—	—
≥65	58	17.1	(16.6, 32.3)	—	—	—
**Histology**						
ADE	71	21.0	(17.5 31.5)	—	—	—
SqCC	67	18.1	(17.2, 29.7)	—	—	—
**Tumor location**						
left	62	22.3	(20.4, 32.0)	28	28.5	(22.5, 35.1)
Right	76	25.1	(13.9 30.2)	33	31.0	(24.2, 36.6)
**Stage category**						
Stage I	35	30.2	(18.9, 36.5)	22	32.5	(28.5, 40.3)
Stage II	39	27.8	(23.1, 44.3)	19	34.0	(24.4, 42.2)
Stage III	42	15.3	(13.4, 36.7)	16	14.0	(13.4, 30.2)
Stage IV	22	12.0	(10.2. 25)	1	46	—
**Tobacco use**						
Smoker	58	17.3	(15.6, 29.0)	—	—	—
**Replase**				—	—	—
Recurrence	62	20.1	(17.5 29.5)	—	—	—

Note.— *Stage is missing for 3 patients from the original data source.

-Indicates the information is hidden by the data source.

Abbreviations: OS, overall survival; CI, confidence interval; Ade, adenocarcinoma; SqCC, squamous cell carcinoma.

**Table 3:** The representative features selected by SVM for histopathology and clinical TNM staging prediction, the highest scored features, and the prognostic values (HR and P value by univariate Cox analysis) of each feature for overall survival.

	Prognosis					
	Pathology	T1/T2-T3/T4	N0/N1-N2/N3	I/II-III/IV	Mean (±SD)*	HR (*P* value)
Texture						
Long Run Emphasis of RL3 (HL)	T				4.93 (7.32)	0.79 (0.212)
Long Run Low Gray Level Emphasis of RL3 (HL)	T				3.41 (3.16)	0.79 (0.227)
Long Run High Gray Level Emphasis of RL3 (HL)	T				1.29 (2.04)	0.71 (0.072)
Long Run Emphasis of RL2 (LH)		T			8.34 (10.5)	1.01 (0.956)
Short Run Emphasis of RL3 (HL)	T	T	T		8.33 (0.79)	1.15 (0.469)
Long Run High Gray Level Emphasis of RL2 (LH)		T			1.96 (2.27)	0.87 (0.506)
Long Run Low Gray Level Emphasis of RL2 (LH)		T			4.50 (5.34)	0.82 (0.298)
Energy of RL1 (LL)		T		T	10.5 (2.36)	0.79 (0.226)
Energy of RL3 (HL)	T		T	T	2.14 (4.62)	0.69 (0.052)
Correlation of CO [2, 2] (LH)		T	T	T	5.11 (3.13)	1.60 (0.004)^+^
Correlation of CO [3, 3] (HL)		T	T	T	2.53 (1.76)	1.48 (0.015)^+^
Correlation of CO [3, 2] (HL)		T	T		2.46 (9.69)	1.54 (0.016)^+^
Correlation of CO [1, 2] (LL)			T		7.83 (1.62)	1.45 (0.052)
Contrast of CO [1, 1] (LL)			T	T	5.29 (2.01)	1.40 (0.511)
Variance of CO [2, 1] (LH)		T			1.81 (0.85)	1.28 (0.850)
Gabor						
PTREntropy of Gabor [1, 11] (LL)	T		T		−4.63 (0.44)	1.13 (0.527)
MTRvariance of Gabor [1, 23] (LL)		T			3.49 (1.58)	1.00 (0.965)
PTRentropy of Gabor [1, 5] (LL)			T		−1.05 (1.09)	0.78 (0.212)
MTRvariance of Gabor [1, 25] (LL)			T		3.13 (2.32)	0.89 (0.542)
MTRmean of Gabor [1, 29] (LL)		T			3.60 (1.19)	1.09 (0.666)
MTRmean of Gabor [1, 25] (LL)		T			6.70 (1.46)	1.21 (0.568)
PTRentropy of Gabor [1, 7] (LL)		T			−4.63 (0.56)	1.10 (0.900)
Shape						
Compactness	T			T	−3.83 (1.97)	1.03 (0.945)
Skewness of HL	T				−0.85 (11.9)	1.03 (0.871)
Skewness of LH		T			−3.65 (8.92)	0.90 (0.588)
Kurtosis of LL		T			13.8 (3.73)	0.58 (0.145)
Kurtosis of HH			T		9.03 (2.03)	1.27 (0.209)
Sphericity				T	1.52 (0.27)	1.08 (0.887)

Note.— T means that the feature is significantly associated (Top-ranked).

^*^Data are mean ± standard deviation, with range in parentheses for normally distributed data.

^+^Indicates a significant difference.

Abbreviations: SD, standard deviation; HR, hazard ratio; RL, run length; CO, co-occurrence; PTR, Gabor phase-based texture representation; MTR, Gabor magnitude texture representation.

**Table 4:** Multivariate Cox-proportional hazards regression analysis of clinical and imaging parameters on the primary cohort and the TCIA cohort (validation set).

Multivariate Cox-proportional hazards regression analysis of radiomics features and prognosis				
Variable	Primary cohort^†^		Validation cohort^‡^	
	HR (95% CI)	P Value	HR (95% CI)	P Value
Age (y)	1.39 (0.85, 1.21)	0.176	—	—
Sex, men	0.72 (0.42. 1.86)	0.178	—	—
Smoke	1.67 (0.80, 2.59)	0.586	—	—
LH-Correlation of CO	1.65 (1.41, 2.75)	0.010^+^	2.74 (1.10, 6.85)	0.027^+^
HL-Correlation of CO	1.75 (1.15, 2.58)	0.007^+^	1.40 (0.63, 3.57)	0.135
N0/N1 vs. N2/N3	0.52 (0.31, 0.87)	0.010^+^	—	—
T1/T2 vs. T3/T4	0.61 (0.34, 1.07)	0.386	—	—
I/II vs. III/IV	0.64 (0.39, 0.85)	0.043^+^	0.30 (0.12, 0.69)	0.006^+^
Ade vs. SqCC	1.52 (0.85, 2.73)	0.152	—	—

Note.— Data in parentheses are 95% confidence intervals.

^†^Model is adjusted for age, sex, tobacco use, tumor position, and clinical TNM staging.

^‡^Model is adjusted for overall clinical stage and tumor position.

^+^Indicates a significant difference.

—Indicates the information is unavailable on the TCIA open access database.

Abbreviations: HR, hazard ratio; CI, confidence interval; CO, co-occurrence; Ade: adenocarcinoma; SqCC, squamous cell carcinoma.
