# Supplementary material for: Non-small cell lung cancer: quantitative phenotypic analysis of CT images as a potential marker of prognosis
Source: Sci Rep. 2016 Dec 6;6:38282. doi: 10.1038/srep38282 (PMC5138817; doi:10.1038/srep38282)
Supplement: Supplementary Materials [file srep38282-s1.pdf]

Non-small cell lung cancer: quantitative phenotypic analysis of CT images as a potential marker of prognosis

Jiangdian Song, PhD Zaiyi Liu, MD, PhD Wenzhao Zhong, MD, PhD Yanqi Huang, MD
Zelan Ma, MD Di Dong, PhD Changhong Liang, MD, PhD Jie Tian, PhD

Appendix A1

A detailed flowchart of this study is presented in Supplementary Fig. S1. The trial of clinical prediction and prognosis were performed successively according to the features extracted from CT images. Segmentation results (GGO, solid, and cavity) were presented in Supplementary Fig. S2. In our Radiomics approach, 592 phenotypic descriptors were extracted from the pre-therapy CT images, mainly 3D, texture, Gabor, and wavelet features. The construction of feature set was presented in Supplementary Fig. S3. Based on the SVM, the features were scored and the highest scored were selected for prediction and following overall survival analysis of NSCLC. Here we presented the radiomics features at the top of the score list, which were divided into four groups: Texture, Gabor, Shape and Wavelet, as described in the manuscript. We introduced the computational formulas of these features in detail in the following paragraphs. In the main text, the number reported behind each feature in Table 3 denoted the image after wavelet transform, the number of 1, 2, 3, and 4 represents the LL, LH, HL and HH image, respectively. In addition, the number of [*x*,*y*] in gray-level co-occurrence matrix: *x* denotes the LL, LH, HL and HH image, *y* represents the different directions: 1, 2, 3 and 4 denote the direction of 0°, 45°, 90° and 135°. The construction of our feature set is described in Supplementary Fig. S3.

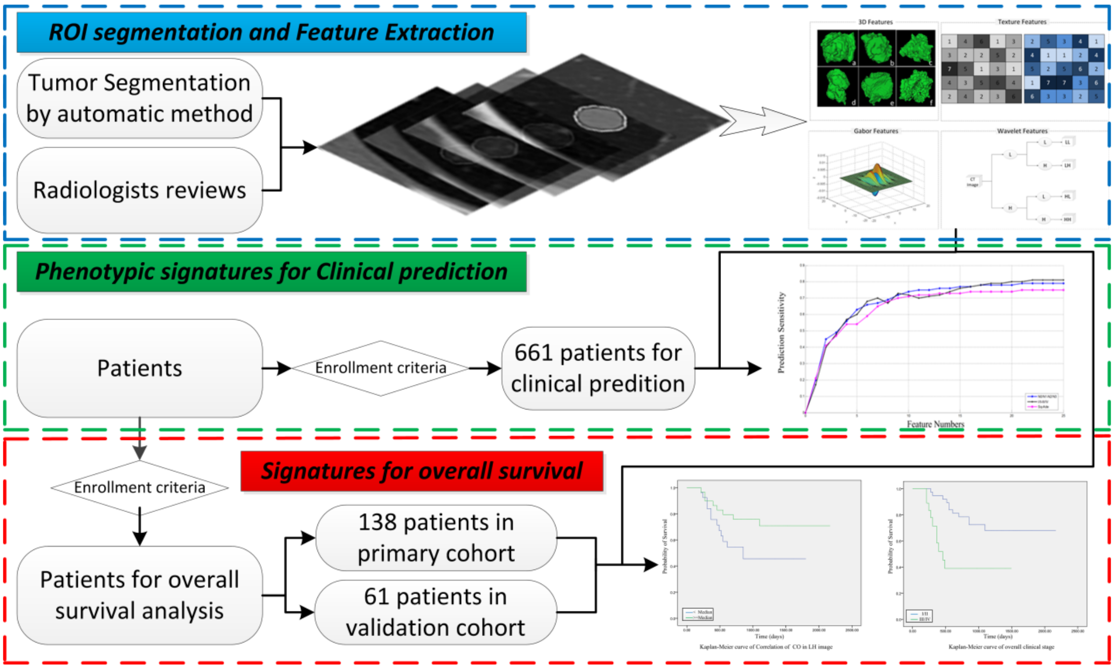

Supplementary Fig. S1. Flowchart of this study: the section of ROI segmentation, feature extraction, and patient

enrollment. The clinical prediction and prognosis are performed successively according to the phenotypic features.

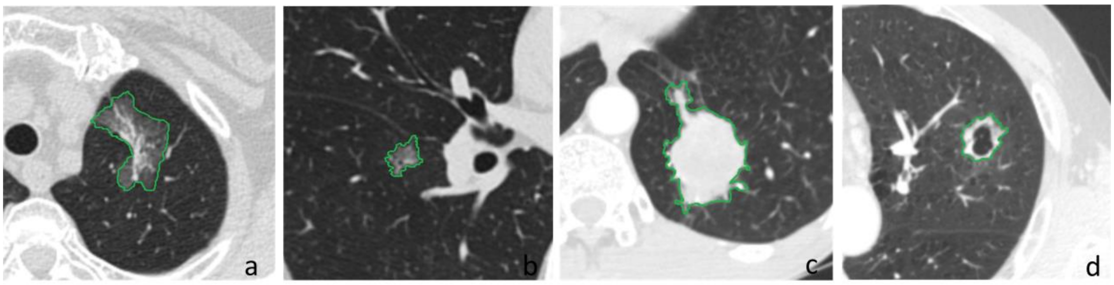

Supplementary Fig. S2. (a) The segmented result of a ground glass opacity (green line labelled) in cross section. non-enhanced CT image in a 52-year-old man with NSCLC (stage T2N0M0). (b) The segmented result of a ground glass opacity (green line labelled) in cross section. non-enhanced CT image in a 65-year-old man with NSCLC (stage T1N0M0). (c) The segmented result of squamous cell carcinoma (green line labelled) in cross section. Contrast-enhanced CT image in a 61-year-old man with NSCLC (stage T2aN2M0). (d) The segmented result of squamous cell carcinoma with cavity (green line labelled) in cross section. Contrast-enhanced CT image in a 60-year-old man with NSCLC (stage T1N0M0).

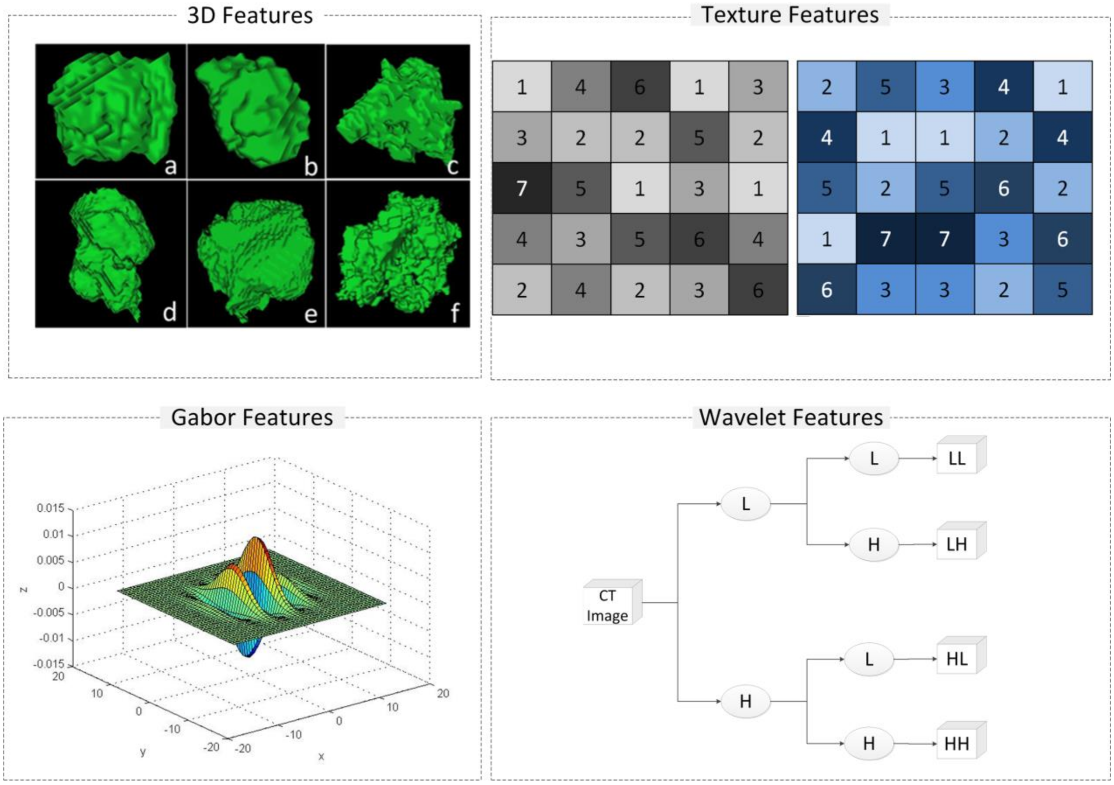

Supplementary Fig. S3. Construction of feature set. The radiographic feature set includes 3D features, Texture features, Gabor features, and Wavelet features.

Group 1: Texture

Run-length is a metrics to quantify gray level runs in an image. Since the consecutive pixels that have the same gray level value in one direction could be measured, the gray level run is defined as the length in number of pixels. In a gray level run length matrix $M(i, j \mid \theta)$, the (i, j) th element describes the number of times the j gray level i appears consecutively in the direction specified by θ. The other definitions are described as follow:

$M(i, j \mid \theta)$ is the (i, j) th point in the given run-length matrix M for a direction θ,

$N(g)$ is the number of discrete intensity values in the image,

$N(r)$ is the number of different run lengths,

$N(p)$ is the number of voxels in the image.

(1) Long Run Emphasis of RL2 and RL3

$$LRE = \frac{\sum_{i=1}^{N(g)} \sum_{j=1}^{N(r)} M(i, j \mid \theta) j^2}{\sum_{i=1}^{N(g)} \sum_{j=1}^{N(r)} M(i, j \mid \theta)}$$

(2) Long Run Low Gray Level Emphasis of RL2 and RL3

$$LRLGLE = \frac{\sum_{i=1}^{N(g)} \sum_{j=1}^{N(r)} [\frac{M(i, j \mid \theta) j^2}{i^2}]}{\sum_{i=1}^{N(g)} \sum_{j=1}^{N(r)} M(i, j \mid \theta)}$$

(3) Long Run High Gray Level Emphasis of RL2 and RL3

$$LRHGLE = \frac{\sum_{i=1}^{N(g)} \sum_{j=1}^{N(r)} M(i, j \mid \theta) j^2 i^2}{\sum_{i=1}^{N(g)} \sum_{j=1}^{N(r)} M(i, j \mid \theta)}$$

(4) Short Run Emphasis of RL3

$$SRE = \frac{\sum_{i=1}^{N(g)} \sum_{j=1}^{N(r)} [\frac{M(i, j \mid \theta)}{j^2}]}{\sum_{i=1}^{N(g)} \sum_{j=1}^{N(r)} M(i, j \mid \theta)}$$

(5) Energy of RL1 and RL3

$$\text{Energy} = \sum_{i=1}^{N(g)} \sum_{j=1}^{N(g)} [M(i, j \mid \theta)]^2$$

The gray-level co-occurrence matrix is defined as $M(i, j, D, \theta)$, a matrix to describe the gray level distribution by a distance of D pixels in direction θ of an image with the size of $N(g) \times N(g)$, where the (i, j) th element represents the number of times the combination of intensity levels occurs in two pixels in the image. The other definitions are described as follows:

$M(i, j)$ is the co-occurrence matrix by the D and θ ,

$N(g)$ is the number of discrete intensity levels in the image,

χ is the mean of $M(i, j)$,

χ_x is the mean of m_x,

χ_y is the mean of m_y,

σ_x is the standard deviation of m_x,

σ_y is the standard deviation of m_y,

(6) Correlation of CO[2,2] and CO[3,3]

$$Correlation = \frac{\sum_{i=1}^{N(g)} \sum_{j=1}^{N(g)} ijM(i,j) - \chi_i(i)\chi_j(j)}{\sigma_x(i)\sigma_y(i)}$$

(7) Contrast of CO[1,1]

$$Contrast = \sum_{i=1}^{N(g)} \sum_{j=1}^{N(g)} |i-j|^2 M(i,j)$$

(8) Variance of CO[2,1]

$$Variance = \sum_{i=1}^{N(g)} \sum_{j=1}^{N(g)} (i-\chi)^2 M(i,j)$$

Group 2: Gabor

Gabor filter, named after Dennis Gabor, is a linear filter used for edge detection, which is usually used in the field of face recognition. It could select valuable image information in different directions and different scales. Therefore, the visual characteristics of the lung lesion could be well described by Gabor filters. We used eight directions and five scales to extract Gabor features. Mean, variance, and entropy were used to construct the Gabor feature group. The number of [*x*,*y*] in Gabor Feature: *x* means the LL, LH, HL and HH image. Gabor magnitude texture representation (GMTR) and Gabor phase-based texture representation (GPTR) are captured using the convolution between multi-scale and multi-directional Gabor wavelet function, and the *y* means the feature we selected on the image in one direction and one scale. Here we use eight directions and five scales so the number of images after Gabor transform is 80 (40 GMTR and 40 GPTR). Nl indicates the length of histogram of gray-level of Gabor image, $P(i)$ denotes the number of gray level i, N indicates the sum of image pixels and the $X(i)$ presents the intensity of i on the Gabor image.

(9) Mean of Gabor

$$Mean = \frac{1}{N} \sum_{i}^{N} X(i)$$

(10) Variance of Gabor

$$Variance = \frac{1}{N-1} \sum_{i}^{N} \left(X(i) - Mean \right)^2$$

(11) Entropy of Gabor

$$Entropy = \sum_{i=1}^{Nl} P\left(i\right)\log_2 P\left(i\right)$$

Group 3: Shape

The shape features present the distribution of voxel intensities on the CT image. The three-dimensional tumor image is X with N voxels. V denotes the volume and A is the surface area of the volume of interest. The followings are the computational formulas of the representative features:

(12) Compactness

$$Compactness = 36\pi \frac{V^2}{A^3}$$

(13) Skewness of HL and LH

$$skewness = \frac{\frac{1}{N}\sum_{i=1}^{N}(X(i) - \bar{X})^3}{(\sqrt{\frac{1}{N}\sum_{i=1}^{N}(X(i) - \bar{X})^2})^3}$$

(14) Kurtosis of LL and HH

$$kurtosis = \frac{\frac{1}{N}\sum_{i=1}^{N}(X(i) - \bar{X})^4}{(\sqrt{\frac{1}{N}\sum_{i=1}^{N}(X(i) - \bar{X})^2})^2}$$

(15) Sphericity

$$sphericity = \frac{\pi^{\frac{1}{3}}(6V)^{\frac{2}{3}}}{A}$$

Appendix A2

The consistency of the highest scored signatures from different readers was tested. We used two radiologists with more than ten years of experience in clinical diagnosis to draw the initial seed point for automatic tumor segmentation. They were mainly responsible for seed point choice, and their selection of seed points was completely independent. Manual segmentation would be performed by clinicians once the automatic segmentation results were poor. We randomly selected 20 cases and the similarity index of features from different segmentation results was presented in Supplementary Fig. S4. The average stability of features from different segmentation results was 92.50%. The inter-class correlation coefficient (ICC) by two observers was ranged from 0.810 to 0.936.

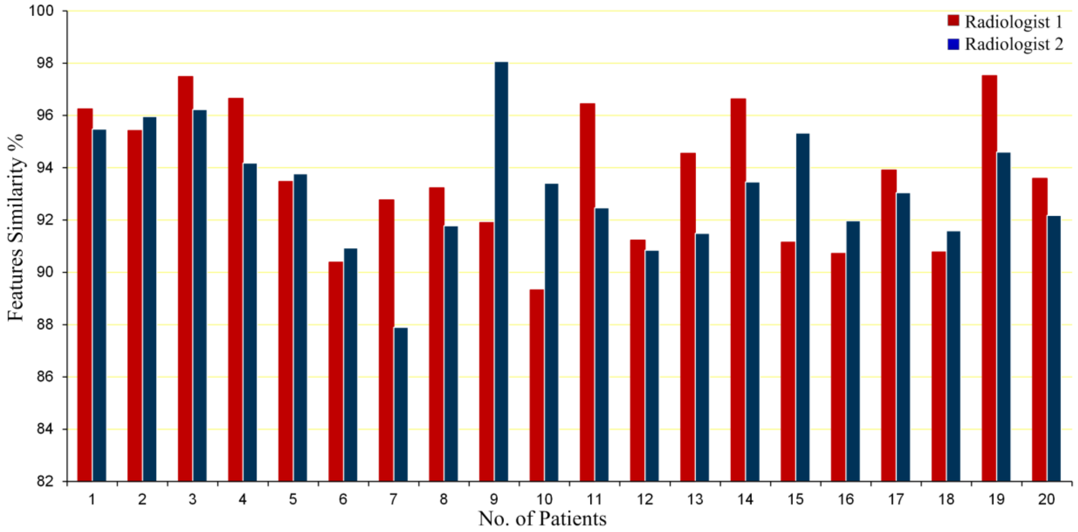

Supplementary Fig. S4. Stability of features on 20 tumors by multiple auto-segmentation from different radiologists. The average stability of each feature is higher than 92.50%.

Appendix A3

5-fold cross-validation process: *K*-fold cross-validation is a universal method for validation in the field of statistics. In this study *5*-fold cross-validation was used. The original sample was randomly partitioned into *5* subsamples. Of the *5* subsamples, a single subsample was retained as the validation data for testing the model, and the remaining 4 subsamples were used as training data. The 5-fold cross-validation process was then repeated 5 times (the number of *folds*), with each of the *5* subsamples used exactly once as the validation data. The results from the all the repeat experiments then been averaged to produce a final estimation. The *5*-fold cross-validation was a good model through a lot of experiments using large data sets and different learning techniques. The advantage of this method over repeated random sub-sampling is that all observations are used for both training and validation, and each observation is used for validation exactly once.

Appendix A4

The response receiver operating characteristic curves (ROC) indicated that the highest scored features selected by SVM could play a significant role for prediction. We tested the predictive ability of the top features for N staging (stage N0/N1 vs. N2/N3), histopathology (squamous cell carcinoma vs. adenocarcinoma), and overall clinical stage (stage I/II vs. III/IV). The area under curve (AUC) of ROC of N staging was 0.79, and the curve of histopathology and overall clinical stage were 0.76 and 0.84. Supplementary Fig. S5 was the predictive performances of the highest scored signatures for histopathology and clinical TNM staging. And Supplementary Figure S6 was the ROC curve of histopathology.

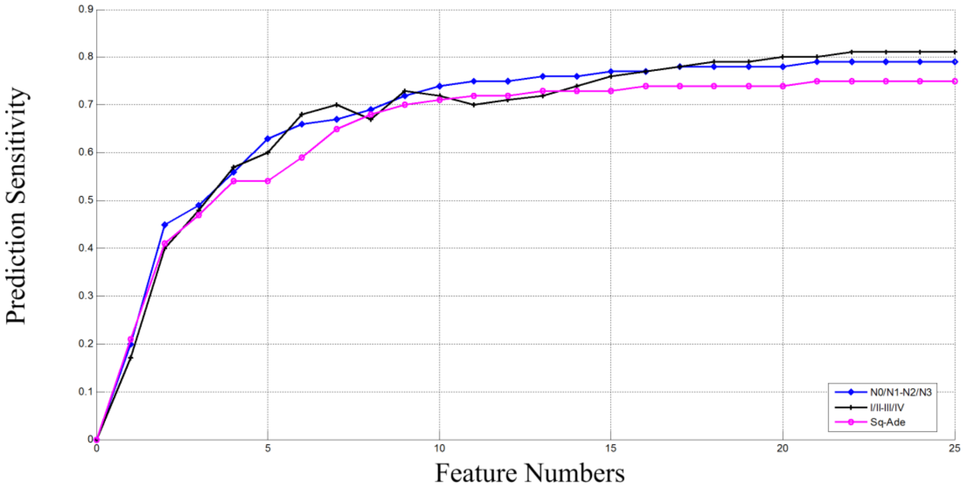

Supplementary Fig. S5. Predictive performances of the top scored signatures for histopathology and clinical TNM staging. The curves present the prediction accuracy of N staging (stage N0/N1 vs. N2/N3), overall clinical stage (stage I/II vs. III/IV), and histopathology (squamous cell carcinoma vs. adenocarcinoma) by different representative features.

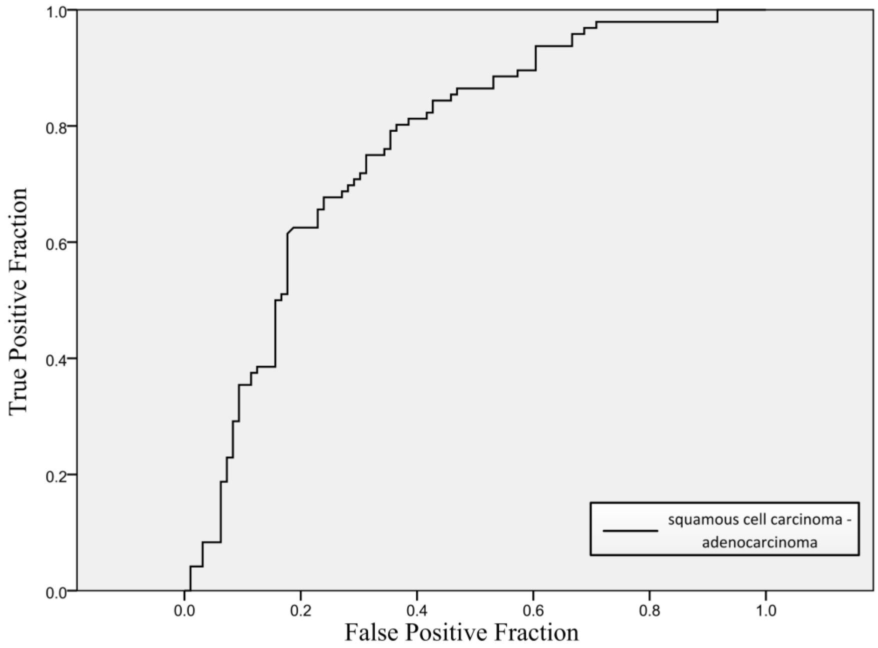

Supplementary Fig. S6. The receiver operating characteristic curve of histopathology prediction on squamous cell carcinoma vs. adenocarcinoma when using the top scored 25 features. The area under the curve is 0.76.
